# The relationship between adverse childhood experiences and emotional eating behaviors among college students: mediated by perceived stress and depression

**DOI:** 10.3389/fpsyg.2025.1698804

**Published:** 2026-03-19

**Authors:** Yi Xia, Weihong Xu, Ruitao Yao, Yan Liang

**Affiliations:** 1 Center for Faculty Development and Research, Guangzhou Medical University, Guangzhou, Guangdong, China; 2 School of Health Management, Guangzhou Medical University, Guangzhou, Guangdong, China; 3 Independent Researcher, Guangzhou, Guangdong, China

**Keywords:** adverse childhood experiences, depression, emotional eating behaviors, mediation analysis, perceived stress

## Abstract

**Background:**

Adverse childhood experiences (ACEs) are well-established risk factors for various mental health issues, including emotional eating behaviors. However, the mediating roles of perceived stress and depression in the relationship between ACEs and emotional eating behaviors, especially among college students, remain underexplored. This study aimed to investigate these associations, with a specific focus on the mediating effects of perceived stress and depression.

**Methods:**

Using a stratified cluster random sampling method, this study recruited 984 students from a university in Guangzhou, China, between May and November 2024. Data were collected on participants' sociodemographic characteristics, perceived stress, depressive symptoms, ACEs, and emotional eating behaviors through self-reported measures. Mediation analysis was conducted using the PROCESS macro (SPSS 4.0) developed by Hayes, controlling for gender, age, place of residence, per capita monthly family income, and family type.

**Results:**

The overall mean score for emotional eating behaviors among college students was 26.45 (*SD* = 12.046), with significant differences observed by sex, educational level, and self-reported health status. ACEs, perceived stress, depression, and emotional eating behaviors were significantly positively correlated (*r* = 0.105–0.473, *P* < 0.01). Although ACEs did not directly predict emotional eating behaviors (95% CI: −0.221–0.740, *P* > 0.05), they influenced emotional eating through two indirect pathways: (1) the independent mediating effect of depression, which accounted for 57.74% of the total indirect effect (β = 0.511, 95% CI: 0.323–0.723, *P* < 0.05), and (2) the serial mediation effect of perceived stress and depression, which accounted for 12.65% of the total indirect effect (β = 0.112, 95% CI: 0.054–0.181, *P* < 0.05).

**Conclusions:**

The findings indicate that depression and perceived stress fully mediate the relationship between ACEs and emotional eating behaviors. Interventions focusing on stress management and emotional regulation may help alleviate the negative impact of ACEs, ultimately promoting healthier eating behaviors among college students.

## Introduction

Adverse Childhood Experiences (ACEs), defined as exposure to abuse, neglect, or household dysfunction prior to age 18, constitute well-established predictors of lifelong health disparities across both somatic and psychiatric domains (Felitti et al., 2019). Accumulating evidence suggests that ACEs predispose individuals to dysregulated eating behaviors in adulthood (Strine et al., 2012), with a pronounced tendency toward emotion-driven food consumption characterized by hedonic appetite regulation rather than homeostatic need (Evers et al., 2010). This maladaptive eating pattern exhibits robust associations with adiposity accumulation, obesity-related comorbidities and cardiometabolic dysregulation (Fuemmeler et al., 2009), thereby compounding allostatic load in vulnerable populations. Elucidating the psychobiological mechanisms underlying emotional eating thus represents a critical step toward developing targeted interventions to attenuate its pathogenic sequelae.

College students, as a transitional group, are particularly vulnerable to emotional eating. They face unique challenges, including academic pressure, interpersonal conflicts, and the need for social adaptation, which can all intensify psychological stress. Neurodevelopmental research indicates that the incomplete myelination of the prefrontal cortex during adolescence (Casey and Jones, 2008) may lead to limited cognitive control, making them more reliant on intuitive coping strategies in emotionally charged situations; for instance, engaging in emotional eating behaviors (Frayn, 2018). Moreover, eating often serves as an escape from negative stimuli, helping individuals avoid direct confrontation with negative self-awareness and shield themselves from information that might threaten their self-image (Litwin et al., 2017). However, this reliance on food for emotional regulation carries significant risks. Research consistently links frequent emotional eating to adverse physical health outcomes, such as weight gain, obesity, metabolic syndrome, and an increased risk of cardiovascular disease (van Strien et al., 2013). Furthermore, it can create a detrimental psychological cycle where negative emotions trigger overeating, which in turn fuels feelings of guilt, shame, and further distress, potentially worsening the original emotional state (Reichenberger et al., 2018).

Given the serious consequences of emotional eating (Hudson et al., 2007), the study sought to explore the relationship between ACEs and emotional eating behaviors. Few empirical research have investigated how individual's early life environment influences emotional eating, and the underlying psychological mechanisms remain unclear. Furthermore, most studies focus on specific populations such as children or obese people, neglecting the sensitive group of college students who are navigating a transitional phase in their lives. Therefore, this study aims to explore the association between ACEs and emotional eating behaviors, as well as the mediating roles of the perceived stress and depression, which is crucial for developing targeted intervention measures and enhancing the health of this group.

## Literature view and research hypothesis

### ACEs and emotional eating behaviors

ACEs are significantly positively correlated with emotional eating behaviors in adulthood. Individuals who have experienced ACEs are more likely to develop Post-Traumatic Stress Disorder (PTSD), and other trauma-related disorders such as depression, anxiety, substance use, impulse control disorders, and personality disorders, which are closely related to emotional eating behaviors in adulthood (Brewerton, 2022). Proffitt Leyva (2018)'s research also indicated that early stressful environments would induce unpredictability schema in individuals, altering body awareness and leading to eat in the absence of hunger. Additionally, studies have found that adolescents who have experienced ACEs such as domestic violence and mental illness are more prone to developing bulimia nervosa, an extreme form of emotional eating behavior (Chu et al., 2022). Therefore, emotional eating behaviors may be related to ACEs. Based on the above analysis, we propose the first hypothesis:

H1: ACEs positively predict emotional eating behaviors.

### Mediating role of perceived stress

According to the Stress-Vulnerability Model, after experiencing early adversity, an individual's emotion regulation ability and stress coping mechanism may be affected in the long term, leading to adverse psychological and behavioral consequences (Zubin and Spring, 1977). Perceived stress, as an individual's cognitive assessment of stressors in the environment, is an important mediating variable connecting early adversity and subsequent behavior (Lazarus, 1984). Studies have shown that ACEs significantly increase an individual's sensitivity to stress, which may disrupt emotion regulation, prompting individuals to use emotional eating to alleviate negative emotions (Evers et al., 2010; Shonkoff and Garner, 2012). Dilsiz and Arslan (2023)'s research on obese individuals similarly indicates that prolonged exposure to stress of obesity stigma weakens self-efficacy, leading to emotional eating. In addition, the mediating role of perceived stress between ACEs and emotional eating has been supported by some studies. For example, individuals with ACEs are more likely to exhibit emotional eating behaviors under stress, closely related to perceived stress levels (Michopoulos et al., 2015). Therefore, perceived stress may be the key psychological mechanism by which ACEs affect emotional eating behaviors. Based on the above analysis, we propose the following hypothesis:

H2: ACEs affect emotional eating behaviors through the mediating role of perceived stress.

### Mediating role of depression

Depression may play a key mediating role in the relationship between adverse childhood experiences (ACEs) and emotional eating behavior. Studies have shown that ACEs significantly increase the risk of depression in adulthood (Nelson et al., 2017). Depression is not only a mood disorder but also linked to emotion regulation impairment, which may lead to overeating to relieve negative emotions (Metin et al., 2024). According to the Emotion Regulation Theory, depressed individuals often lack effective emotion regulation strategies and are more inclined to emotional eating (Stice et al., 2002; Gross, 1995). At the same time, ACEs may further aggravate the frequency and intensity of emotional eating behavior by inducing long-term depressive symptoms. Therefore, depression may be an important psychological mechanism by which ACEs affect emotional eating behavior. Based on the above analysis, we propose the following hypothesis:

H3: ACEs affect emotional eating behaviors through the mediating role of depression.

### Chain mediating role of perceived stress and depression

Based on the Stress-Emotion Regulation Theory, adverse childhood experiences (ACEs) may affect emotional eating behavior through multiple psychological mechanisms. First, ACEs significantly increase an individual's sensitivity to stress, making them more likely to experience high levels of perceived stress in adulthood (Wang et al., 2023). This persistent state of stress may further induce emotion regulation disorders and increase the risk of depression (Wang et al., 2024). Depression, an emotional disorder, is closely linked to impaired emotion regulation, prompting individuals to use eating as a means of emotional relief (van Strien, 2018). Therefore, perceived stress and depression may form a chain mediation path between ACEs and emotional eating behavior. Specifically, ACEs increase perceived stress levels, worsening depressive symptoms. Depressed individuals, lacking effective emotion regulation, are more prone to emotional eating as a coping mechanism (Goldschmidt et al., 2014). Based on the above analysis, we propose the following hypothesis:

H4: ACEs affect emotional eating behaviors through the chain mediating effects of perceived stress and depression.

**Figure 1:**
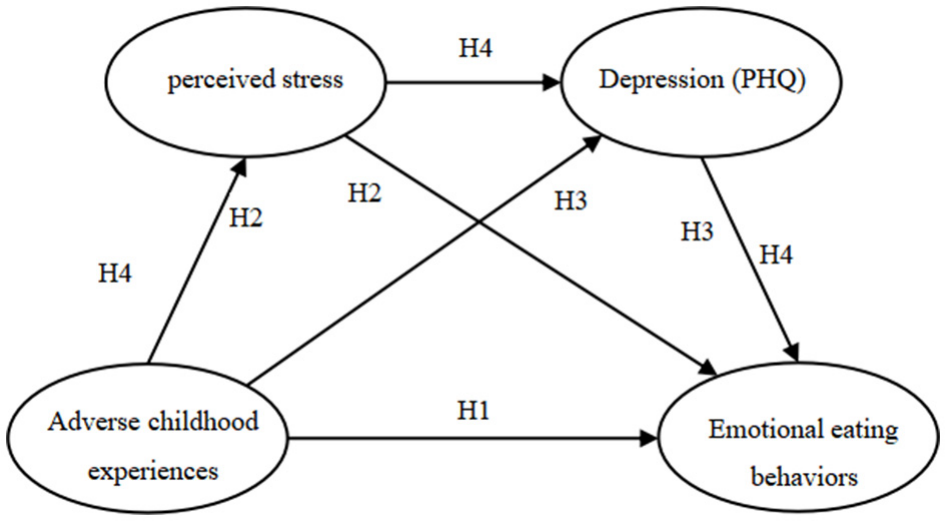
Multiple mediating hypothesis model between variables. H1, Adverse childhood experiences → Emotional eating behaviors; H2, Adverse childhood experiences → perceived stress → Emotional eating behaviors; H3, Adverse childhood experiences → Depression (PHQ) → Emotional eating behaviors; H4, Adverse childhood experiences → perceived stress → Depression (PHQ) → Emotional eating behaviors.

This study mainly explored the relationship between ACEs and emotional eating behavior among college students. Stress and depression were used as key mediating variables through a chain mediation model (Figure 1) to reveal their mechanism of action between ACEs and emotional eating behavior. This study enhances our understanding of how ACEs influence eating behaviors and provides empirical evidence to inform targeted interventions that may mitigate the long-term health consequences associated with ACEs.

## Method

### Study design

From July to October 2024, this cross-sectional survey was conducted at two different campuses of a medical university in Guangzhou. After a pilot survey to revise the questionnaire based on undergraduate and graduate students' feedback, a stratified cluster sampling design was adopted. In this study, classes were grounded by grade, and 1–2 classes were randomly selected from each group. The inclusion criteria were voluntary participation (informed of the study and providing consent), being a currently enrolled full-time student, and adequate Chinese language skills. Exclusion criteria included incomplete questionnaires and illogical response. The Ethics Committee of Guangzhou Medical University approved the study (Approval No.: 202406003).

### Participants

Ultimately, we collected a total of 1,035 questionnaires, with a response rate of 98.7%. After identifying and deleting data with missing values and logical problems (e.g., selecting the same answer five or more times consecutively, or choosing options inconsistent with trap questions). The final valid samples were 984, meeting the priori sample size requirement (*n* > 530), with an effective recovery rate of 95.5%. This survey was conducted through offline paper questionnaires and online questionnaires on the www.wjx.cn platform. Before distributing questionnaires, participants were fully informed about the study's objective, completion guidelines, and their anonymous, voluntary participation. Research assistants were on-site during the survey to address questions and ensure smooth completion and data reliability.

### Measurements

#### Adverse childhood experiences

ACEs were assessed using the Chinese version of the Revised Adverse Childhood Experience Questionnaire (ACEQ-R; Chen et al., 2022), a 14-item instrument developed through cross-cultural adaptation of Finkelhor's expanded framework (Finkelhor et al., 2015). Compared with the original 10-item ACE Questionnaire (ACEQ), the ACEQ-R adds peer victimization, social exclusion, community violence exposure, and socioeconomic deprivation to the original three domains (abuse, neglect, and household dysfunction; Copeland et al., 2013). The scale measures adverse experiences of participants before the age of 18, with no occurrences counted as “0” and occurrences counted as “1.” The total ACEQ-R score of an individual is obtained by accumulating the scores of the 14 items on the scale (range: 0–14), and score greater than 1 indicates having experienced ACEs. The higher the total score, the more types of negative experiences have been experienced. In this study, Cronbach's α coefficient for the scale was 0.718.

#### Perceived stress

To assess individuals' perception of life stress, Cohen et al. (1983) developed the Perceived Stress Scale (PSS). Yang and Huang (2003) translated and revised PSS based on the cultural background of China, forming the Chinese version of the Perceived Stress Scale (CPSS). The scale includes 14 items, with seven items reflecting a sense of tension and seven items assessing a sense of loss of control. The Likert five-point scale is used to score the CPSS, ranging from 1 (never) to 5 (always). Total scores of 0–28 indicate normal stress levels, 29–42 indicate elevated stress, and 43–56 indicate excessive stress. It is a widely used stress perception measurement tool in China. In the study, the Cronbach's α coefficient for the CPSS was 0.824.

#### Depression

The Patient Health Questionnaire-9 (PHQ-9), a widely used self-report instrument developed by Kroenke et al. (2001), was employed to assess the severity of depression symptoms in participants over the past 2 weeks. The PHQ-9 consists of nine items, each corresponding to a specific symptom of depression. Participants rate the frequency of these symptoms on a four-point Likert scale, ranging from 0 (“not at all”) to 3 (“nearly every day”), and the total scores is 27. Higher total scores on the PHQ-9 indicate greater severity of depressive symptoms. A total score of 10 or more is often used as a cut-off for identifying individuals with clinically significant depression (Kroenke et al., 2001). The PHQ-9 is well-validated and has demonstrated good reliability across a variety of populations, including among college students (Levis et al., 2019). In the current study, the Cronbach's α coefficient for the PHQ-9 was 0.852.

#### Emotional eating behaviors

In this study, emotional eating was assessed using the Emotional Eating Subscale from the Dutch Eating Behavior Questionnaire (DEBQ) developed by Strien et al. (1986). The DEBQ is one of the most widely used tools in the research of emotional eating behavior, with established reliability and validity. The Emotional Eating Subscale used in this study consists of 13 items designed to evaluate individuals' eating behaviors in various negative emotional states. The scale employs a five-point Likert scale, ranging from 1 (never) to 5 (often) and the total score is 65. Higher scores reflect more pronounced emotion-induced eating behaviors. This subscale has been widely used in multiple countries and has also demonstrated good applicability in China (Ye et al., 2020). In the current study, the Cronbach's α coefficient of this scale was 0.956.

### Data analyses

In this study, we strive to select well-established scales with high reliability and anonymous measures. Statistical analyses were conducted using IBM SPSS 25 (IBM Corp.), with two-tailed tests. Data normality was assessed using the Kolmogorov–Smirnov normality test, and the results met the criteria for normality. The *t*-test was used for continuous variables, and the one-way ANOVA were used for categorical variables to compare the differences. In the subsequent hypothesis test, use the collinearity test to check whether the inflation factor of the variable exceeds 10 to indicate whether there is collinearity between the variables. Moreover, the Harman single-factor test was used to assess the common method bias. Pearson's correlation coefficient were performed to investigate the associations among ACEs, emotional eating behaviors, perceived stress and depression. Using the PROCESS plugin in SPSS (Model 6) to examine the relationship between the mediating effect of perceived stress and depression between adverse childhood experiences and emotional eating behavior was evaluated. The analysis controlled for gender, age, residence, family monthly per capita income, and family type, and bias correction and accelerated Bootstrap confidence intervals (95% BCa CIs) were used to correct bias and skewness. It was considered statistically significant if the confidence interval did not include the value 0 for the adverse childhood experiences on emotional eating Behaviors. Finally, we obtained the total, direct, and indirect mediating effects of each variable.

## Result

### Descriptive statistics and analysis of differences

In this study, a total of 984 students met the eligibility criteria, which included 317 males (32.2%) and 667 females (67.8%). An independent-samples *t*-test revealed that the total score for emotional eating behaviors significantly differed between males and females (*t* = −2.022, *p* < 0.05), with females prefer to emotional eating behaviors. Regarding educational level distribution among the students, the data are presented in Table 1, with 478 freshman (48.6%), 246 sophomore (25%), 140 junior (14.2%), 41 senior (4.2%), and 79 students at the master's or doctoral level One-way ANOVA and *post-hoc* tests indicated that ACEs had a more pronounced impact on students in the master/doctor and senior classes compared to those in the freshman class (*F* = 4.447, *p* < 0.01). Furthermore, freshman students exhibited a higher likelihood of engaging in emotional eating behaviors relative to their sophomore and junior counterparts (*F* = 4.003, *p* < 0.01). These findings, as detailed in Table 1, highlight the significant differences in ACEs and emotional eating behaviors across different educational levels. In terms of family type, the distribution is shown in Table 1. Statistical analysis revealed that ACEs had a greater impact on students from single parent families and reorganized families than on those from core families (*F* = 16.438, *p* < 0.001). In terms of self-reported health status, the distribution is shown in Table 1, with 57.4% reporting good, 37.6% medium, and 5.0% poor. One-way ANOVA and *post-hoc* tests revealed that students with better self-reported health status were less likely to experience ACEs and emotional eating. What's more, 139 students (14.1%) self-reported chronic disease and 845 students (85.9%) self-reported no chronic disease. An independent-samples *t*-test revealed that the total score for ACEs significantly differed between this group (*t* = 4.521, *p* < 0.001), students with chronic diseases have higher rates of ACEs (Table 1).

**Table 1:** Descriptive statistics and tests for differences in ace and emotional eating behaviors based on different demographic characteristics (*N* = 984).

**Variables**	**ACE**			**Emotional eating behaviors**	
	***N*** **(%)**	***M*** ±***SD***	***t/F***	***M*** ±***SD***	***t/F***
**Sex**					
Male	317 (32.2%)	0.97 ± 1.582	−1.134	25.32 ± 12.040	−2.022^*^
Female	667 (67.8%)	1.09 ± 1.709		26.98 ± 12.021	
**Residence**					
Urban	743 (75.5%)	1.02 ± 1.649	−0.989	26.35 ± 11.900	−0.466
Rural	241 (24.5%)	1.15 ± 1.732		26.76 ± 12.503	
**The only child**					
Yes	222 (23.6%)	1.12 ± 1.773	0.652	25.15 ± 12.113	−1.830
No	762 (77.4%)	1.03 ± 1.639		26.83 ± 12.007	
**Educational level**					
Freshman	478 (48.6%)	0.86 ± 1.575	4.447^**^	27.44 ± 12.548	4.003^**^
Sophomore	246 (25%)	1.23 ± 1.655		26.96 ± 12.622	
Junior	140 (14.2%)	1.05 ± 1.611		23.38 ± 10.114	
Senior	41 (4.2%)	1.39 ± 2.072		23.22 ± 8.929	
Master/doctor	79 (8.0%)	1.53 ± 1.973		26.00 ± 10.618	
**Family per capita monthly income (CNY)**					
≥10,000	210 (21.3%)	0.95 ± 1.515	1.323	26.77 ± 12.154	0.815
8,000–9,999	110 (11.2%)	1.05 ± 1.791		25.80 ± 11.367	
5,000–7,999	277 (28.2%)	0.94 ± 1.665		26.61 ± 12.116	
3,000–4,999	240 (24.4%)	1.13 ± 1.601		25.55 ± 11.151	
< 3,000	147 (14.9%)	1.29 ± 1.884		27.63 ± 13.591	
**Family type**					
Core family	899 (91.4%)	0.94 ± 1.563	16.438^***^	26.56 ± 12.073	0.902
Single parent family	60 (6.1%)	2.15 ± 2.154		24.92 ± 10.275	
Reorganized family	21 (2.1%)	2.57 ± 2.599		27.38 ± 15.743	
Intergenerational family	4 (0.4%)	1.00 ± 1.414		19.00 ± 7.118	
**Self-reported health status**					
Good	565 (57.4%)	0.79 ± 1.385	32.075^***^	26.36 ± 11.254	8.448^***^
Medium	370 (37.6%)	1.26 ± 1.853		27.40 ± 12.548	
Bad	49 (5.0%)	2.57 ± 2.151		31.82 ± 14.924	
**Chronic diseases**					
Yes	139 (14.1%)	1.64 ± 2.029	4.521^***^	28.00 ± 12.790	3.790
No	845 (85.9%)	0.96 ± 1.583		26.19 ± 11.907	

ACE, Adverse childhood experiences, ^*^p < 0.05, ^**^p < 0.01, ^***^p < 0.001.

### Correlation analysis between variables

The results of Pearson correlation coefficients of ACEs, perceived stress, Depression and Emotional Eating behaviors are presented in Table 2. Results show that ACEs, perceived stress, Depression and Emotional Eating behaviors are positively related to each other.

**Table 2:** Correlation matrix of adverse childhood experiences, perception of pressure, depression (PHQ), and emotional eating behaviors.

**Variable**	***M***	***SD***	**1**	**2**	**3**	**4**
1. Adverse childhood experiences	1.06	1.669	1			
2. Perceived stress	22.86	8.156	0.169^**^	1		
3. PHQ	4.89	3.865	0.368^**^	0.473^**^	1	
4. Emotional eating behaviors	26.45	12.046	0.105^**^	0.117^**^	0.248^**^	1

^**^p < 0.01 (2-tailed).

**Figure 2:**
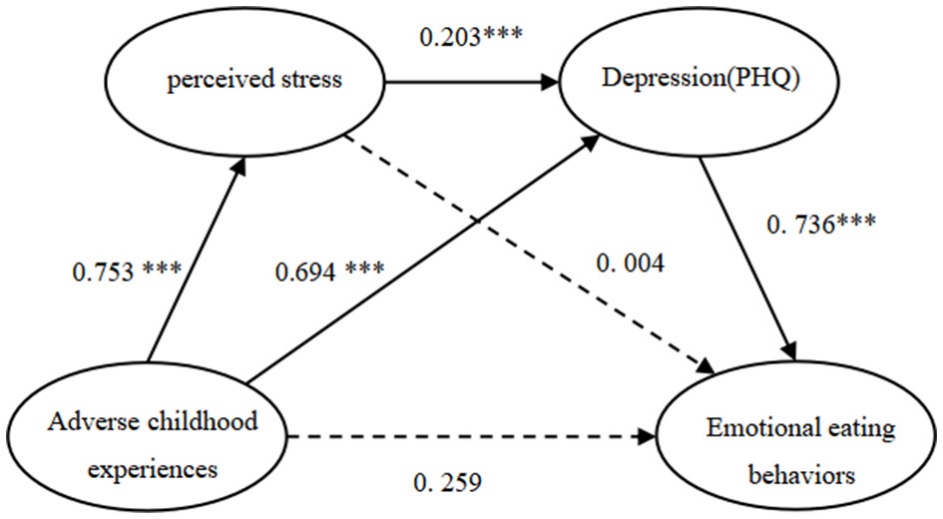
The multiple mediation of perceived stress and depression between adverse childhood experiences and Emotional eating behaviors. ^***^p < 0.001.

A common method bias test indicated that the first factor without rotation was 20.375%, below the 30% critical threshold (Podsakoff et al., 2003), suggesting the absence of significant common method bias. Then emotional eating behaviors were the dependent variable, while ACEs were taken as the independent variable, perceived stress, and depression were the mediating variables. As illustrated in Table 3 and Figure 2, ACEs positively related to perceived stress (β = 0.753, *p* < 0.001) and depression (β = 0.753, *p* < 0.001). Perceived stress was positively related to depression (β = 0.203, *p* < 0.001). Depression was positively related to emotional eating behaviors (β = 0.736, *p* < 0.001). The two paths within the research model were statistically significant, supporting hypotheses 3 and 4. The results showed that ACEs did not directly predict emotional eating behaviors (95% CI [−0.221, 0.740]), but the path through depression was significant. Moreover, a significant chain mediating effect of perceived stress and depression was observed.

**Table 3:** Regression analysis of the chain mediation model.

**Variable**	**Perceived stress**		**Depression (PHQ)**		**Emotional eating behaviors**	
	β	***P***	β	***P***	β	***P***
ACE	0.753	< 0.001	0.694	< 0.001	0.259	>0.05
Perceived stress			0.203	< 0.001	0.004	>0.05
Depression (PHQ)					0.736	< 0.001
*R* ^2^	0.207		0.316		0.074	
*F*	7.256		64.481		9.784	

Results of standardize estimate, direct effect and model summary information for the parallel multiple mediator model are presented in Table 4. The total indirect effect of ACEs through perceived stress, and depression on emotional eating behaviors was statistically significant (β = 0.626; 95% CI [0.414, 0.865]), accounted for 70.73% of the total effect ratio. When considering the mediating variables individually and in combination, both the single mediation depression (β = 0.511; 95% CI [0.323, 0.723]), and the serial-multiple mediation of perceived stress and depression (β = 0.112; 95% CI [0.054, 0.181]) were found statistically significant, and, respectively, accounted for 57.74% and 12.65% of the total effect ratio.

**Table 4:** Standardization effect and direct effect in the model.

**Path**	**Standardized coefficient**	***P***	**95% confidence interval**		**Ratio of effect (%)**
			**Lower**	**Upper**	
H4: ACE → perceived stress → depression (PHQ) → emotional eating behaviors	0.112	< 0.05	0.054	0.181	12.65
H3: ACE → depression (PHQ) → emotional eating behaviors	0.511	< 0.05	0.323	0.723	57.74
H2: ACE → perceived stress → emotional eating behaviors	0.003	>0.05	−0.080	0.084	0.34
Total indirect effect	0.626	< 0.05	0.414	0.865	70.73
H1: direct effect	0.259	>0.05	−0.221	0.740	29.27
Total effect	0.885	< 0.05	0.426	1.345	

ACE, Adverse childhood experiences.

## Discussion

This study revealed that there are significant gender differences in emotional eating behavior, with females exhibiting higher scores than males. This finding is consistent with previous research, suggesting that women are more likely to use eating as a coping mechanism when confronted with negative emotions (Mantzios, 2014). From a physiological standpoint, hormonal changes in women may influence the regulation of emotions and appetite, thereby increasing the frequency of emotional eating (Gibson, 2006). Psychologically, women may place greater emphasis on body image and self-evaluation, making them more prone to using eating as a means of emotional regulation during times of emotional fluctuation (Valero-García et al., 2021). Additionally, sociocultural factors may also impact women's emotional eating behavior. For example, societal constraints on emotional expression among women may lead them to seek psychological comfort through eating (Johnson et al., 2012).

The research findings indicate that there are significant differences in emotional eating behavior among students of different grades. Emotional eating shows a U-shaped pattern across academic stages. It is more common among entry-level students (e.g., undergraduates) and advanced students (e.g., postgraduates), while students in the middle stages show lower levels of this behavior. This may be related to the academic stress, social environment, and psychological developmental stages that students face at different grade levels. Students in lower grades may be more influenced by their family environment and face the transition stress from high school to university. Studies have shown that adolescents and emerging adults are more likely to adopt unhealthy coping strategies when facing stress, such as emotional eating (Gardner and Steinberg, 2005). It was found that students in higher grades, especially postgraduates, are more prone to emotional eating due to greater academic pressure and uncertainty about future career prospects (Grajek et al., 2022). The lower scores in emotional eating behavior among middle-grade students (juniors/seniors) may be because they have gradually adapted to the academic and social environments of university life and have relatively stronger emotional regulation abilities. Research has shown that emotional regulation abilities in college students gradually increase with grade level, which may help reduce the occurrence of emotional eating (Jiang, 2025). These factors may support why higher prevalence of emotional eating behavior among students in lower and higher grades compared with those in middle grades.

Studies have found that students who self-report lower levels of health are more likely to engage in emotional eating behaviors. This may be because students with poorer health status lack effective coping strategies to manage emotional distress, leading them to rely more on eating as an emotional regulation mechanism (Lazarus, 1984). In addition, differences in health awareness and coping strategies may also lead to this result. Students who self-report higher levels of health may pay more attention to healthy lifestyles, have better emotion regulation abilities and coping strategies, and are therefore less likely to engage in emotional eating behaviors (Shonkoff and Garner, 2012).

### The mediating role of perceived stress and depression

Childhood abuse, as an adverse psychological trauma experience, has a profound impact on an individual's mental health and behavior patterns. In recent years, more and more studies have begun to focus on the relationship between childhood abuse and emotional eating. Studies have shown that there is a significant positive relationship between childhood abuse and emotional eating. Specifically, individuals who suffered childhood abuse are more likely to engage in emotional eating behaviors in adulthood. A number of empirical studies have supported the positive impact of childhood abuse on emotional eating. Mantzios (2014) found that individuals who suffered psychological abuse in childhood often find it difficult to properly deal with negative emotions when faced with problems because their parents provide less emotional support, and are more likely to engage in emotional eating or binge eating. Kong and Bernstein (2009)'s research pointed out that survivors of childhood abuse may turn to self-destructive and self-harming behaviors in the absence of internal self-soothing ability, such as purging and vomiting to relieve tension and distress and regulate their internal emotional state, which is also an extreme manifestation of emotional eating. However, this study found that the direct effect of adverse childhood experiences (ACEs) on emotional eating behavior was not significant (95% CI [0.221, 0.740]). This suggests that the effect of adverse childhood experiences on emotional eating behavior may be achieved through other mediating variables. This result is consistent with the study of Michopoulos et al. (2015), who found that the effect of childhood trauma on emotional eating was mainly achieved through mediating factors such as emotion regulation disorders.

The results of this study showed that depression played a full mediating role between adverse childhood experiences and emotional eating behavior (β = 0.511, 95% CI [0.323, 0.723]). This suggests that adverse childhood experiences may correlated with depressive symptoms in adults, and depressive symptoms associated with the high frequency of emotional eating behavior (Stice et al., 2002). Adverse childhood experiences have been widely proven to be closely related to depressive symptoms in adulthood (Berens et al., 2017). These adverse experiences may be associated with heightened emotional regulation disorders in adults, thereby increasing the risk of depression (Nelson et al., 2017). According to emotion regulation theory, individuals with depression often lack effective emotion regulation strategies and are therefore more likely to cope with negative emotions through emotional eating (Gross, 1995). Further pointed out that improving emotion regulation ability can effectively reduce emotional eating behavior (Keng et al., 2011). At the same time, the results of this article are consistent with the findings of Ghaffari et al. (2024) and Proffitt Leyva (2018), the hypothesis H3 in this study was supported. Which showed that depression played a mediating role in the relationship between adverse childhood experiences and emotional eating behavior. Therefore, intervention measures should focus on improving emotion regulation ability and help individuals master healthier coping strategies to reduce the occurrence of emotional eating behavior.

However, the mediating effect of perceived stress between adverse childhood experiences and emotional eating was not supported in this sample (β = 0.003, 95% CI [−0.080, 0.084]). Although there is a certain correlation between stress and emotional eating, it is not a direct causal relationship. Some literature points out that perceived stress itself does not directly affect emotional eating, but indirectly affects eating behavior by inducing mediating variables such as depression and anxiety (Michopoulos et al., 2015). Additionally, highly stressed individuals may adopt different coping strategies around eating, such as Sedatic Hunger behaviors—only driven by biological need without the pursuit of taste or emotion (Arslan et al., 2025). Therefore, when exploring the relationship between stress and emotional eating, it is necessary to consider the influence of other variables.

### Chain-mediating role of perceived stress and depression

This study also found that perceived stress and depression had a chain mediation effect between adverse childhood experiences and emotional eating behavior (β = 0.112, 95% CI [0.054, 0.181]), the hypothesis H4 in this study was supported. Adverse childhood experiences (ACEs) have been widely shown to be closely related to the ability to perceive stress in adulthood. These adverse experiences may increase the sensitivity of individuals to stressors in adulthood, making them more likely to experience high levels of perceived stress. Shonkoff and Garner (2012) pointed out that adverse childhood experiences may lead to long-term emotional regulation disorders and increase the risk of perceived stress in adulthood. This increase in perceived stress may further affect the individual's mental health and behavioral patterns. The results of this study showed a significant positive correlation between perceived stress and depression. Increased perceived stress may correlated with higher emotional regulation disorders, which in turn increases the risk of depression. Nelson et al. (2017) found through meta-analysis that there was a significant positive correlation between childhood abuse and depressive symptoms in adulthood, and perceived stress played a mediating role in this relationship. The results of this study also show that there is a significant positive correlation between depression and emotional eating behavior. This is consistent with the results of Evers et al. (2010). Depressed individuals lack effective coping strategies when facing negative emotions, and may choose to relieve their emotions through emotional eating.

Individuals with adverse childhood experiences are more sensitive to negative emotions and stress due to trauma, and their cognition and behavior are more limited, making them more likely to develop depression than ordinary people. Because they are more sensitive to stressful events, individuals often need to consume more self-control in the process of emotional regulation. This high intensity of emotional regulation may trigger their unpleasant memories and emotional reactions to childhood traumatic experiences. In the face of these negative emotions, emotional eating often seems to be used as the important coping mechanism in life.

### Practical implications

The results of this study provide a theoretical basis for designing intervention measures for emotional eating behavior among college students. Given the mediating role of depression and perceived stress between adverse childhood experiences and emotional eating behavior, intervention measures should focus on emotion regulation and stress management. For example, mental health education courses can be used to help students master effective emotion regulation strategies, such as cognitive behavioral therapy and mindfulness meditation (Kabat-Zinn, 1990). In addition, providing psychological counseling services and support groups to help students cope with the psychological trauma caused by adverse childhood experiences can also help reduce the occurrence of emotional eating behavior (Brewerton, 2022).

This study has several limitations. First, reliance on self-reported measures of perceived stress, depression and emotional eating behaviors may introduce self-report biases and recall biases. Second, the cross-sectional design precludes establishing causality between ACEs, perceived stress, depression, and emotional eating behaviors. Third, the study focused only on stress and depression as mediators, while other potential factors (e.g., social support, self-esteem) were not explored. Lastly, the sample was limited to one university. The single-site sampling may limits generalizability to other Chinese contexts. To address these limitations, future research should: (a) Combine self-reports with objective measures (e.g., cortisol levels for stress); (b) Use longitudinal designs to track mediation processes over time; (c) Test supplementary integrative models incorporating social, psychological, and biological mediators; (d) Replicate findings in diverse cultural and age cohorts to assess contextual moderators.

## Conclusion

This study investigated the mediating mechanisms linking adverse childhood experiences (ACEs) to emotional eating behaviors among 984 Chinese college students. While ACEs showed no direct association with emotional eating, mediation analysis revealed two significant indirect pathways: (1) depression as an independent mediator and (2) a sequential chain through perceived stress leading to depression. These findings underscore that stress and depressive symptoms fully mediate the impact of ACEs on maladaptive eating patterns, suggesting interventions targeting emotional regulation and stress resilience may mitigate such behavioral risks. Future research should validate these pathways across diverse populations and develop context-specific psychological interventions.

## Data Availability

The raw data supporting the conclusions of this article will be made available by the authors, without undue reservation.
